# Job burnout among Chinese university counselors: a meta-analysis study

**DOI:** 10.3389/fpsyg.2026.1872378

**Published:** 2026-08-17

**Authors:** Cheng Yang, Jingyuan Tian, Rui Jiang, Jason H. Huang

**Affiliations:** 1 Department of Psychology, Sichuan Normal University, Chengdu, China; 2 School of Basic Medical Sciences, Chengdu University of Traditional Chinese Medicine, Chengdu, Sichuan, China; 3 Department of Neurosurgery, Baylor Scott & White Health Center, Temple, TX, United States

**Keywords:** depersonalization, emotional exhaustion, job burnout, meta-analysis, reduced personal accomplishment, university counselors

## Abstract

**Objective:**

To study the current state of the three dimensions of emotional exhaustion, depersonalization, and reduced personal accomplishment among Chinese university counselor(s), and to determine whether there are differences based on different conditions such as measurement instruments, regions, and publication years.

**Methods:**

We comprehensively searched PubMed, Web of Science, CNKI, Wanfang Data, and VIP from 2000 to March 2026. We included cross-sectional studies that assessed job burnout among Chinese university counselors with the Modified Maslach Burnout Inventory (MBI) or its validated English and simplified Chinese versions, all built on the original three dimensions of the MBI. In total, 19 studies involving 3,248 participants met our inclusion criteria. Because the studies used Likert scales ranging from 4 to 7 points, we calculated standardized mean differences (SMDs) using the midpoint of each scale for direct comparison. We performed a random-effects meta-analysis in Stata 18.0 and used subgroup analyses together with meta-regression to explore sources of heterogeneity.

**Results:**

The pooled SMDs indicated that Chinese university counselors exhibited the lowest levels of depersonalization with a large effect size (SMD = −1.30), alongside moderate levels of emotional exhaustion (SMD = −0.59) and reduced personal accomplishment (SMD = −0.55). All three dimensions were significantly below the theoretical scale midpoints (all *p* < 0.01). No significant differences were found between the two MBI versions (MBI-ES vs. MBI-GS) or between the Eastern and Central-Western regions. Meta-regression indicated that publication year was significantly associated with increased emotional exhaustion (*β* = 0.154, *p* = 0.048) and depersonalization (*β* = 0.15, *p* = 0.019), showing an increasing trend over time.

**Conclusion:**

Chinese university counselors generally maintain relatively low levels of depersonalization, which represents the most favorable profile among the three burnout dimensions. However, emotional exhaustion and reduced personal accomplishment remain moderate concerns that require attention. To effectively address these issues, universities need to prioritize faculty well-being, optimize workload distribution, and gradually improve the construction of vocational support systems.

## 1 Introduction

Burnout has become one of the most serious occupational health problems in recent years. In 2019, the World Health Organization formally recognized burnout as an occupational phenomenon in the 11th Revision of the International Classification of Diseases (ICD-11), defining it as a syndrome resulting from chronic workplace stress that has not been successfully managed (World Health Organization, 2019). Meanwhile, it has garnered widespread global attention as a critical workplace health concern, drawing increasing interest from public health researchers. In the 1970s, Freudenberger (1974) first proposed that prolonged interpersonal stress among human service workers could lead to burnout. Since then, burnout has been extensively investigated across occupations, with growing research attention paid to professions involving high emotional labor and frequent public contact, including healthcare, education, psychotherapy, and social work (Maslach et al., 2001; Mullen et al., 2018). Growing societal awareness of this issue has coincided with rising work intensity worldwide, leaving many individuals vulnerable to burnout and resulting in substantial personal and workplace impairments. As modern life accelerates and work becomes more psychologically demanding, boundaries between professional and personal life have gradually blurred. Accordingly, researchers and practitioners have shown heightened interest in exploring the mechanisms of burnout and developing targeted interventions (De Sawal et al., 2022; Anderson, 2021).

Maslach and her colleagues (Maslach and Jackson, 1981; Maslach et al., 2001; Maslach and Leiter, 2016) have developed the most widely recognized conceptualization of burnout: a three-dimensional syndrome encompassing emotional exhaustion, depersonalization, and reduced personal accomplishment. This tripartite model has become a global benchmark for burnout assessment and provides the theoretical foundation for the Maslach Burnout Inventory (MBI) and its revised versions, such as the Educators Survey (MBI-ES) and the General Survey (MBI-GS), both of which were adopted in the primary studies included in this meta-analysis. The framework conceptualizes burnout not merely as transient fatigue but as a progressive deterioration of psychological well-being induced by chronic stress. This process undermines work motivation and job performance, while also negatively impacting interpersonal relationships and overall quality of life. Emotional exhaustion refers to emotional depletion/fatigue; depersonalization reflects a detached, cynical attitude toward service recipients; and reduced personal accomplishment denotes diminished feelings of self-efficacy and achievement at work (Maslach and Jackson, 1981; Maslach et al., 2001; Jiang et al., 2022). Although the cross-cultural validity of this model has been verified across diverse societies and occupational settings over the past four decades (Maslach and Leiter, 2016), its applicability within the collectivist cultural context of Chinese higher education remains understudied.

### 1.1 Burnout in higher education and student support roles

Burnout is more likely to occur in the field of higher education among staff involved in extensive contact with students and others, such as student affairs staff, academic advisors, and university counselors (Winfield and Paris, 2022; O’Rourke, 2024; Walker, 2023). The functions of these positions demand continuous emotional support for the psychological, academic, and all-round development of students, amid growing institutional pressure characterized by rising demands for mental health care, heavy administrative workloads, resource scarcity, and higher expectations for crisis intervention and student achievement. Following the end of the pandemic, the demand for mental health support has increased in Western higher education, and staff shortages as well as blurred work-life boundaries have led to burnout among staff members. As a result, turnover intention is relatively high, staff departures occur, and the quality of student support services may be affected (Soria et al., 2026; Winfield and Paris, 2022; O’Rourke, 2024). Based on the above studies, emotional exhaustion has consistently been identified as the primary indicator, with its main causes being continuous emotional labor and role conflict (Anderson, 2021; De Sawal et al., 2022; Mullen et al., 2018). Prolonged high levels of burnout may harm individuals’ health, reduce their enthusiasm for work, and increase the frequency of job changes (Soria et al., 2026; Mullen et al., 2018), thereby weakening the stability of the student life support system. These reasons indicate that a focused study of burnout among higher education support roles is urgently needed, as the well-being of these employees directly affects students’ learning environments, university quality of life, and all-round development. Research worldwide has shown that the proportion of burned-out faculty and student support staff is relatively high, with emotional exhaustion being the most common issue, a situation that exists in many higher education institutions (Jolicoeur, 2025; Knight, 2025).

### 1.2 The unique role and challenges of Chinese university counselors

Counselors in Chinese universities hold a diverse set of professional positions and are closely involved in national education. According to the guidelines of the Central Committee of the Communist Party of China and State Council (2004) on strengthening ideological and political education for college students, counselors are officially positioned as “life mentors” and “trusted friends” of university students. In addition to providing psychological counseling and crisis intervention services, these counselors (as ideological and political education teachers) are also responsible for students’ ideological and political education, daily life management, moral education, class construction, and connecting students with the university administration. With the significant expansion of higher education enrollment—from approximately 7 million students in 1999 to more than 40 million in recent years—the diversity among students has increased, as have mental health problems, and campus crises have become more frequent; thus, the workload of counselors has risen substantially. Many counselors are responsible for the spiritual, educational, psychological, and other life-related issues of hundreds of students at the same time, with long working hours and high pressure. Consequently, role conflict and emotional labor burden have increased for some individuals (Huan, 2025; Sun et al., 2025).

According to the theory of emotional labor (Hochschild, 1983), Chinese counselors need to manage their own emotions to meet the demands of the job. Specifically, this process involves surface acting, which entails suppressing or faking emotions and consequently drains cognitive resources leading to exhaustion. In contrast, deep acting requires consciously modifying inner feelings to align with institutional values, a mechanism that may foster professional identity and buffer against depersonalization (Sun et al., 2025; Liang and Yin, 2025). Although surface acting may increase the risk of emotional exhaustion or depression, deep acting may reduce depersonalization by fostering a strong sense of national mission. Due to these special cultural and institutional requirements, a different type of burnout has emerged in the East compared with the individualistic and transactional nature of professional life in the West. In addition to the higher workload caused by combining ideological education and psychological counseling, many regions also have a high number of students per counselor and scarce institutional resources, thus increasing the difficulties faced by Chinese university counselors. It is necessary to understand the specific features of these problems to design context-appropriate countermeasures and policies that align with the goals of higher education in China and the national strategy of “lifelong learning”.

### 1.3 Research status and gaps

Although the number of studies on job burnout among Chinese university counselors has gradually increased, they are still scattered and lack systematization. The 19 cross-sectional studies included in this meta-analysis (Table 1) were published mainly between 2007 and 2021, and generally showed that the burnout level of counselors is relatively moderate. However, the results of the three main dimensions differed greatly, which was due to differences in the measuring instruments, regions, and data collection times.

**Table 1:** Overview of all enrolled study articles.

First author (year)	Region	Sample Size	Measurement tool (version)	Scoring method	Emotional exhaustion mean ± SD	Depersonalization mean ± SD	Reduced personal accomplishment mean ± SD	AHRQ quality Score
Cao (2007)	Hunan	124	MBI-ES	7-point	3.49 ± 0.63	2.82 ± 0.60	3.26 ± 0.61	7
Jiang and Qin (2008)	Guangxi	162	MBI-GS	5-point	3.75 ± 0.60	3.78 ± 0.53	3.76 ± 0.48	8
Yan (2011)	Anhui	108	Self-developed	5-point	1.26 ± 0.66	1.11 ± 0.63	2.36 ± 0.65	7
Zeng (2011)	Sichuan	129	MBI-ES	5-point	2.23 ± 0.64	1.95 ± 1.01	3.01 ± 0.73	6
Huang and Liu (2011)	Anhui	324	Self-developed	5-point	2.24 ± 0.74	2.18 ± 0.56	2.07 ± 0.76	7
Chen et al (2011)	Hunan	283	MBI-ES	5-point	2.79 ± 0.77	2.06 ± 0.49	2.32 ± 0.39	8
Liang (2013)	Hunan	117	MBI-ES	5-point	1.49 ± 0.70	1.31 ± 0.71	2.40 ± 0.76	7
Liu (2013a)	Guizhou	199	MBI-ES	5-point	2.57 ± 0.68	2.00 ± 0.80	2.46 ± 0.66	8
Chen (2013)	Guangdong	193	Self-developed	5-point	3.20 ± 0.67	1.84 ± 0.58	2.36 ± 0.78	7
Liu (2013b)	Liaoning	60	Self-developed	4-point	2.36 ± 0.79	2.14 ± 0.73	3.45 ± 0.69	6
Zhang (2014)	Henan	124	MBI-GS	7-point	2.60 ± 1.30	2.19 ± 1.24	2.37 ± 1.18	7
Wang (2014)	Shandong	74	MBI-GS	7-point	2.88 ± 1.37	2.25 ± 1.17	2.33 ± 0.84	7
Zhang (2016)	Fujian	127	Self-developed	6-point	2.74 ± 0.98	2.74 ± 0.76	2.14 ± 0.52	8
Liang (2016)	Liaoning	187	MBI-GS	7-point	2.88 ± 1.37	2.25 ± 1.17	3.67 ± 0.82	7
Deng (2017)	Guangdong	463	MBI-ES	4-point	2.21 ± 0.49	1.93 ± 0.48	2.00 ± 0.45	9
Shi (2017)	Sichuan	133	MBI-ES	5-point	2.71 ± 0.45	1.97 ± 0.36	2.77 ± 0.51	7
Guo (2018)	Jiangsu	90	MBI-ES	4-point	2.67 ± 0.54	2.34 ± 0.46	2.79 ± 0.83	7
Yu (2018)	Zhejiang	150	MBI-GS	7-point	3.92 ± 1.43	1.65 ± 1.53	3.32 ± 1.93	7
Yang (2021)	Sichuan	201	MBI-ES	5-point	3.27 ± 0.72	3.62 ± 0.99	3.95 ± 0.46	8

Self-developed = localized/adapted scale based on the three-dimensional MBI framework. (2013a) = Guizhou study; (2013b) = Liaoning study.

Based on the time of publication, the first group of studies (2007–2013), such as those by Cao (2007); Jiang and Qin (2008); Chen et al. (2011); Liang (2016); and (2013), generally used 5-point or 7-point scales. Emotional exhaustion ranged from 2.5 to 3.8, depersonalization was relatively low (1.1–2.8), and reduced personal accomplishment was between 2.0 and 3.5; therefore, previous research has already indicated that emotional exhaustion is a primary cause of stress. Studies in the mid-period (2014–2018), such as those by Deng (2017), Guo (2018), and Shi (2017), used relatively larger samples (100–463 participants) and 4- to 7-point scales. Although there were still some differences in means, the depersonalization scores were generally lower. Recently, other studies (such as Yang, 2021) have reported slightly higher means of 3.27 for emotional exhaustion and 3.62 for depersonalization, which may be due to increased pressure after the COVID-19 pandemic and other changes in counselors’ work demands.

Geographically, research conducted in the eastern coastal areas has generally concluded that reduced personal accomplishment is caused by fierce competition for promotional positions in this highly developed region. On the other hand, research in the central and western inland areas (such as Hunan, Sichuan, Guizhou, Anhui, Guangxi, Liaoning, and Henan) has more frequently reported relatively higher levels of emotional exhaustion; this may be due to resource scarcity and a high student-to-counselor ratio in these areas. Eleven studies employed the MBI-ES as a measurement tool, six used the MBI-GS, and two adapted local scales based on the three-dimensional framework of the MBI. The Likert scales had different numbers of points, so their raw scores could not be directly compared. For example, means from 4-point scales (e.g., Deng, 2017) were generally lower than those from 7-point scales (e.g., Jiang and Qin, 2008; Liang, 2016). Cao (2007) also used reverse scoring for the reduced personal accomplishment dimension, thus increasing the complexity of data integration.

Although the above primary studies have provided some initial information on counselor burnout, they are not without methodological deficiencies: most are limited to a single region, use small or medium-sized samples in cross-sectional designs, lack national representativeness, use inconsistent measurement instruments that have not been culturally validated, focus on demographic characteristics for investigating influencing factors (rarely covering organizational resources or policy effects), and exhibit high heterogeneity, making it difficult for any single study to draw universal conclusions.

From a theoretical perspective, the Job Demands-Resources (JD-R) model (which has been implicitly used in several primary studies, such as Chen, 2013) provides a useful framework: high job demands reduce individuals’ personal resources and cause burnout, whereas job resources can alleviate this negative effect. Emotional labor theory also suggests that Chinese counselors, who need to perform both surface and deep acting in their work with students, may have different burnout patterns compared to their Western counterparts (Sun et al., 2025; Liang and Yin, 2025). Similar frameworks have also been used abroad to study the situation of student affairs professionals, and some common risk and protective factors have been identified (Mullen et al., 2018). However, culture-specific protective factors in China, such as a strong sense of national mission and a collectivist professional identity, have rarely been systematically integrated into empirical research on burnout. Therefore, existing literature does not fully explore the dimensional differences and cross-cultural factors related to counselor burnout.

Moreover, key variables such as regional disparities, temporal trends, and the actual impact of national policies (the 2014 Interim Standards for University Counselors’ Professional Competence and the 2017 Ministry of Education Order No. 43) have not been adequately examined through large-scale synthesis. Most existing research is at a descriptive level, and there is a lack of large-scale synthesis to compare measurement tools, regional differences, and time trends. These problems indicate that a more comprehensive meta-analysis of all related studies is needed to more clearly identify the different types of burnout in this group.

### 1.4 Objectives

The purposes of this study are as follows: (1) to synthesize the existing literature and determine the overall levels of the three dimensions of job burnout among Chinese university counselors; (2) to examine differences in burnout levels across research instruments (MBI-ES and MBI-GS) and geographic regions (eastern versus central-western China) using subgroup analysis; and (3) to explore the moderating effects of publication year, proportion of male participants, and sample size through meta-regression.

To justify these specific analytical choices, we formulated the following rationales based on the JD-R model and the unique socio-cultural context of China. First, comparing the MBI-ES and MBI-GS is essential to verify whether the specific framing of the scale affects burnout reporting. Second, given the significant regional economic disparities in China, we examined the Eastern versus Central-Western regions to explore how regional differences in institutional resources might modulate counselor burnout. Finally, regarding the meta-regression variables, we selected publication year to capture temporal shifts driven by major national policies, male proportion to explore potential gender differences in emotional labor coping, and sample size to assess potential small-study effects.

Building upon these rationales, and based on the JD-R model and emotional labor theory, we formulated specific working hypotheses. We hypothesized that Chinese university counselors would experience moderate emotional exhaustion but relatively lower depersonalization due to cultural factors emphasizing moral education. Furthermore, we expected no significant differences in burnout levels across the MBI-ES and MBI-GS versions. Finally, we posited that publication year would be positively associated with burnout levels, reflecting increasing work demands over time.

## 2 Materials and methods

### 2.1 Search strategy

**Figure 1:**
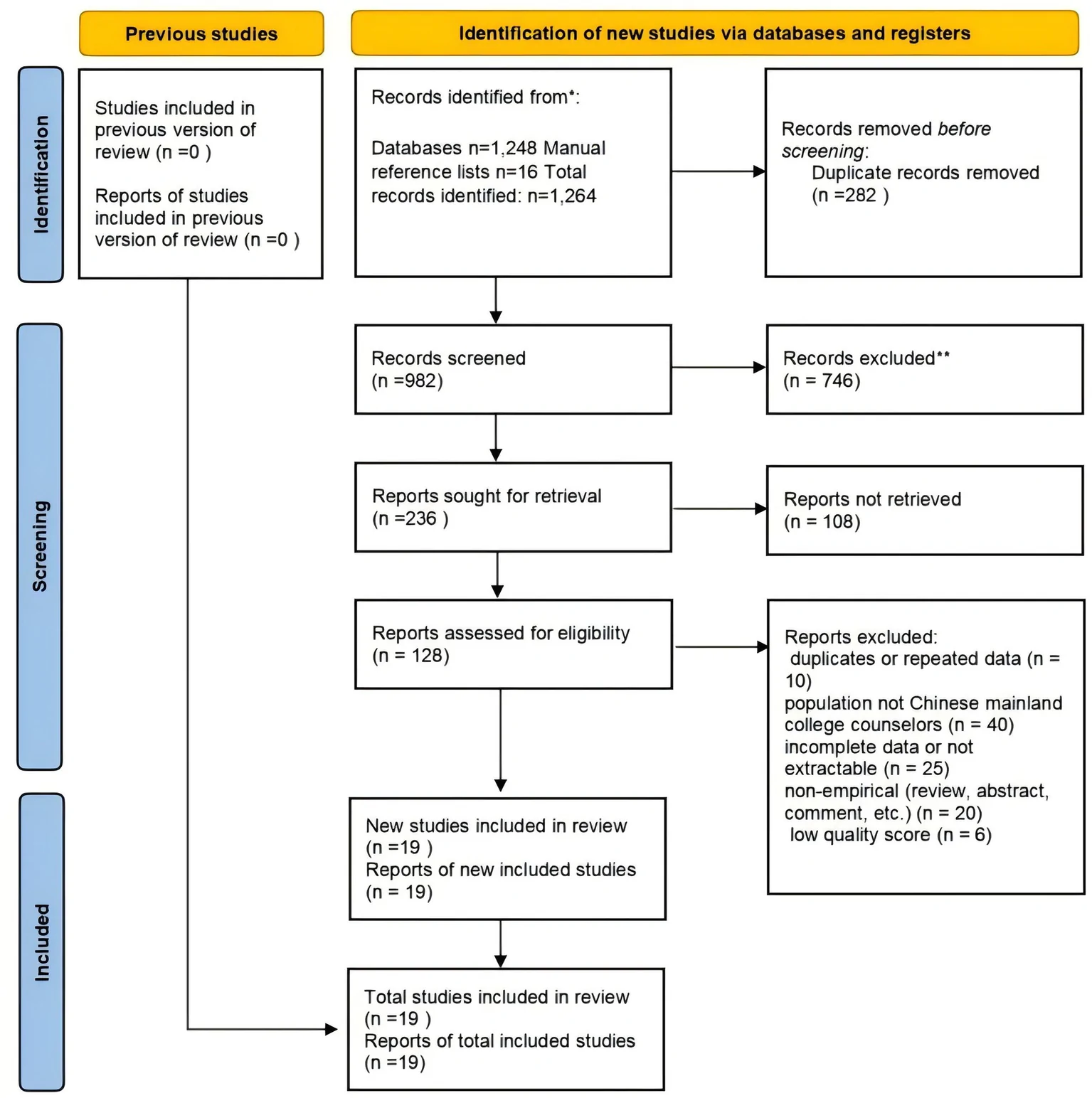
PRISMA flow diagram.

A systematic search was conducted across the PubMed, Web of Science, China National Knowledge Infrastructure (CNKI), Wanfang Data, and VIP databases, covering the period from 2000 to March 2026. Chinese search terms included “高校辅导员” AND (“职业倦怠” OR “情绪耗竭” OR “去个性化” OR “成就感降低” OR “个人成就感”); English terms included (“university counselor” OR “college counselor” OR “student affairs professional”) AND (“job burnout” OR “emotional exhaustion” OR “depersonalization” OR “reduced personal accomplishment” OR “cynicism”). Boolean operators, truncation, and proximity operators were applied when supported by the databases. The reference lists of all the included studies and relevant review articles were manually searched to identify additional eligible publications. Grey literature was not included to maintain methodological standards. All parts of the search were conducted in accordance with the PRISMA 2020 guidelines (Page et al., 2021), and a specific flowchart is shown in Figure 1. A few research teams used this method to avoid language and publication bias and gathered data from both abroad and in China.

### 2.2 Inclusion and exclusion criteria

The inclusion criteria were deliberately stringent to ensure high comparability: (1) participants were full-time Chinese university counselors working in undergraduate institutions, higher vocational colleges, or independent colleges; (2) the study employed a cross-sectional design; (3) the original Maslach Burnout Inventory or its validated revised versions, such as the MBI-ES, MBI-GS, or localized scales explicitly based on the MBI three-dimensional framework, were utilized; (4) means, standard deviations, and sample sizes for all three dimensions (emotional exhaustion, depersonalization, and reduced personal accomplishment) were reported; and (5) the studies were published in Chinese or English in peer-reviewed journals, doctoral dissertations, or master’s theses. Notably, the restriction to cross-sectional designs was adopted because empirical research on counselor burnout in China is overwhelmingly cross-sectional due to practical constraints; thus, focusing on this design was necessary to accumulate a sufficient sample size for robust meta-analytic pooling Chen and Qiu (2006).

The exclusion criteria eliminated duplicate publications or overlapping samples; studies targeting non-university counselor populations, such as secondary school teachers or general administrative staff; studies with incomplete data or an inability to extract and convert the required statistics; non-empirical studies, including reviews, conference abstracts, case reports, and editorials; and studies scoring below 6 on the AHRQ quality assessment tool. Together, these criteria ensured that the synthesis was based on uniform, high-quality quantitative data suitable for robust meta-analytic pooling.

### 2.3 Study selection and data extraction

Two independent researchers (CY and JT) screened the titles, abstracts, and full texts in EndNote 21 software, and a third researcher (RJ) resolved any disagreements by consensus. The reliability of the observers was good (kappa = 0.92). A standardized data extraction form was developed and employed across all included studies. The extracted variables included the first author, publication year, region, sample size, measurement tool and version, Likert-scale scoring method, raw means and standard deviations for each burnout dimension, and the Agency for Healthcare Research and Quality (AHRQ) score. Raw data are presented in tables and figures and are reproduced verbatim. All extracted data were verified for correctness.

### 2.4 Data transformation

Because of the difference in Scale Types 4–7, raw means were converted into standardized mean differences (SMD) using a scale-specific midpoint as the reference point. The Formula was:


$$\mathrm{SMD}=\frac{M{\_}_{\text{onserved}}\_M{\_}_{\text{midpoint}}}{\mathrm{SD}}$$

Where M__midpoint_ = 2.5 (4-point), 3 (5-point), 3.5 (6-point), or 4 (7-point). Negative SMDs indicated scores below the midpoint (lower burnout levels). Many studies have also been based on the reverse scaling of reduced personal accomplishment. For instance, according to Cao (2007), the original mean of reduced personal achievement was 4.74 (SD = 0.61) on a seven-point scale; after being reversed before calculating SMD, it ensured that it directionally matched with the other two dimensions. Previous studies have shown that there is still considerable under-reporting or large discrepancies in the standard deviation among correct MBI data sets for Chinese occupational groups (Wang, 2024). That is, their average scores were relatively low. Therefore, transparent preprocessing steps, including midpoint standardization, were applied. This approach enabled direct comparison across studies while preserving the original variability and theoretical interpretability of the burnout dimensions for future reference.

### 2.5 Quality assessment

The quality of included studies was assessed independently by two raters using the 11-item Agency for Healthcare Research and Quality (AHRQ) instrument for cross-sectional studies Zeng et al. (2015). This instrument evaluates domains including selection bias, study design quality, measurement validity, confounding control, and statistical analysis. Studies scoring below 6 points were considered low quality and excluded; those scoring 6–7 were rated as moderate quality, and those scoring 8–11 were rated as high quality. Overall, the evidence supporting the pooled estimates was regarded as relatively strong to very strong.

### 2.6 Statistical analysis

A meta-analysis was performed using Stata software (version 18.0). Considering the anticipated heterogeneity across studies, a random-effects model with the DerSimonian-Laird estimator was adopted. This estimator was selected as it provides a robust method for calculating the between-study variance, making it highly suitable for observational meta-analyses where substantial heterogeneity is anticipated. Pooled standardized mean differences (SMDs) with their corresponding 95% confidence intervals (CIs) were calculated for each of the three burnout dimensions. Heterogeneity was assessed using Cochran’s Q test and the I^2^ statistic; an I^2^ value greater than 75% was considered to indicate high heterogeneity. Subgroup analyses were conducted according to measurement tool (MBI-ES vs. MBI-GS) and geographic region (eastern vs. central-western China). Meta-regression was performed to explore potential sources of heterogeneity, with publication year, proportion of male participants, and log-transformed sample size as covariates.

Sensitivity analysis was conducted using the leave-one-out method to evaluate the robustness of the pooled estimates by systematically excluding one study at a time. Publication bias, which refers to the phenomenon where studies with statistically significant results are more likely to be published, was evaluated through funnel plots, Egger’s test, and Begg’s test Egger et al. (1997). If asymmetry was detected, the trim-and-fill procedure was applied to adjust the pooled estimates. All statistical tests were two-sided with a significance level of 0.05. The analytical procedures followed best-practice guidelines for meta-analyses of observational studies (Higgins et al., 2003; Thompson and Higgins, 2002; Viechtbauer, 2010).

## 3 Results

### 3.1 Characteristics of included studies

The initial search yielded 1,264 records. After duplicate removal and screening of titles, abstracts, and full texts based on the predefined inclusion and exclusion criteria, 19 studies were ultimately included (see Figure 1 for the PRISMA flow diagram). These studies collectively involved 3,248 Chinese university counselors, providing a reasonably large pooled sample for this specific occupational population in higher education.

As shown in Table 1, the included studies were published between 2007 and 2021 and covered multiple provinces and municipalities in mainland China, spanning economically developed eastern coastal regions (e.g., Shandong, Fujian, Zhejiang, Guangdong, and Jiangsu) and resource-limited central-western regions (e.g., Hunan, Sichuan, Guizhou, Anhui, Guangxi, Liaoning, and Henan). Sample sizes ranged from 60 to 463 participants per study, with a mean of approximately 179 and a median of 150. Of these, 10 studies used the Maslach Burnout Inventory-Educators Survey (MBI-ES), seven used the Maslach Burnout Inventory-General Survey (MBI-GS), and the remaining two employed simplified or adapted versions of the MBI.

Due to differences in Likert-scale formats (4-point vs. 7-point), the raw mean scores across studies varied considerably. For example, emotional exhaustion ranged from 1.26 to 3.92, depersonalization from 1.11 to 3.78, and reduced personal accomplishment from 2.00 to 3.95. To enable direct comparison while preserving original variability, raw scores were converted to standardized mean differences (SMDs) using scale-specific midpoints as reference values.

Quality assessment using the AHRQ Cross-Sectional Studies Assessment Tool indicated that eight studies were of high quality (scores 8–11) and eleven were of moderate quality (scores 6–7); no studies were rated as low quality. Thus, the overall evidence base for the pooled estimates is considered relatively stable and robust. Notably, although the literature search extended to March 2026, no additional eligible cross-sectional studies were identified after 2022, suggesting a potential stagnation or shift in research focus on this topic in recent years.

### 3.2 Meta-analysis of emotional exhaustion

**Figure 2:**
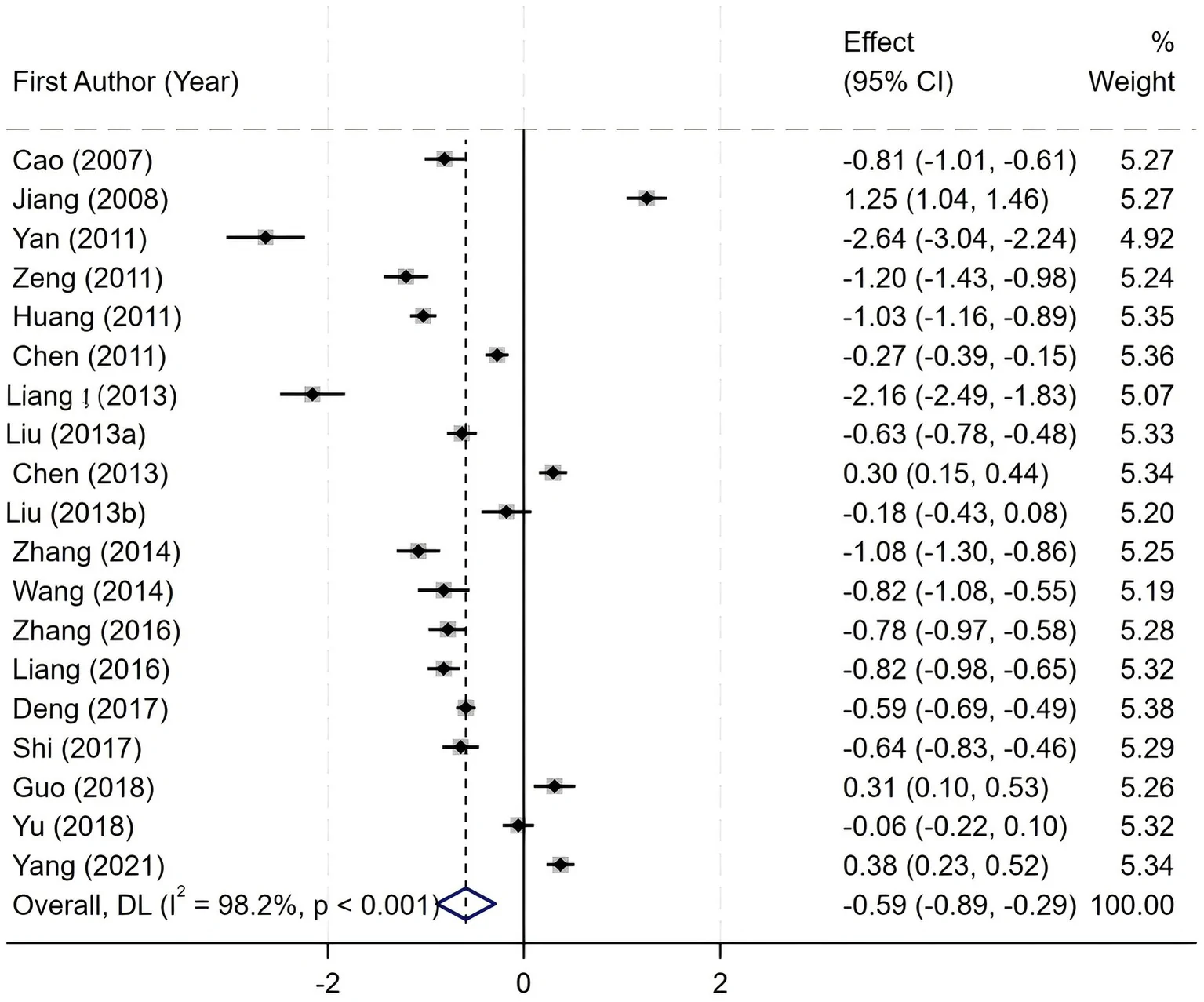
Forest plot for emotional exhaustion.

Random-effects meta-analysis (Figure 2) showed a pooled SMD of −0.59 (95% CI: −0.89 to −0.29) for emotional exhaustion, which was statistically significant (Z = −3.897, *p* < 0.001) and indicated a moderate negative effect size according to Cohen’s conventions (moderate effect typically |SMD| ≈ 0.5). As visualized in the forest plot (Figure 2), most of the 19 individual studies (approximately 16 studies) reported negative SMD point estimates, suggesting that emotional exhaustion levels among Chinese university counselors were generally below the theoretical scale midpoint. The difference in the specific effect size range of this research is significant, many are close to zero, and there is an obvious deviation in the direction. As a result of this kind of design, there was essentially complete statistical heterogeneity (*I*^2^ = 98.2%, Q-test *p* < 0.001); other causes were likely to influence differences: institutional type, counselor duration, student-to-counselor materials, and unobserved organizational stress factors also affected the observed differences. This kind of large disparity commonly exists in many other higher education-based occupational-psychology meta-analysis studies; thus, a follow-up on the moderating effects would become valuable in the future.

### 3.3 Meta-analysis of depersonalization

**Figure 3:**
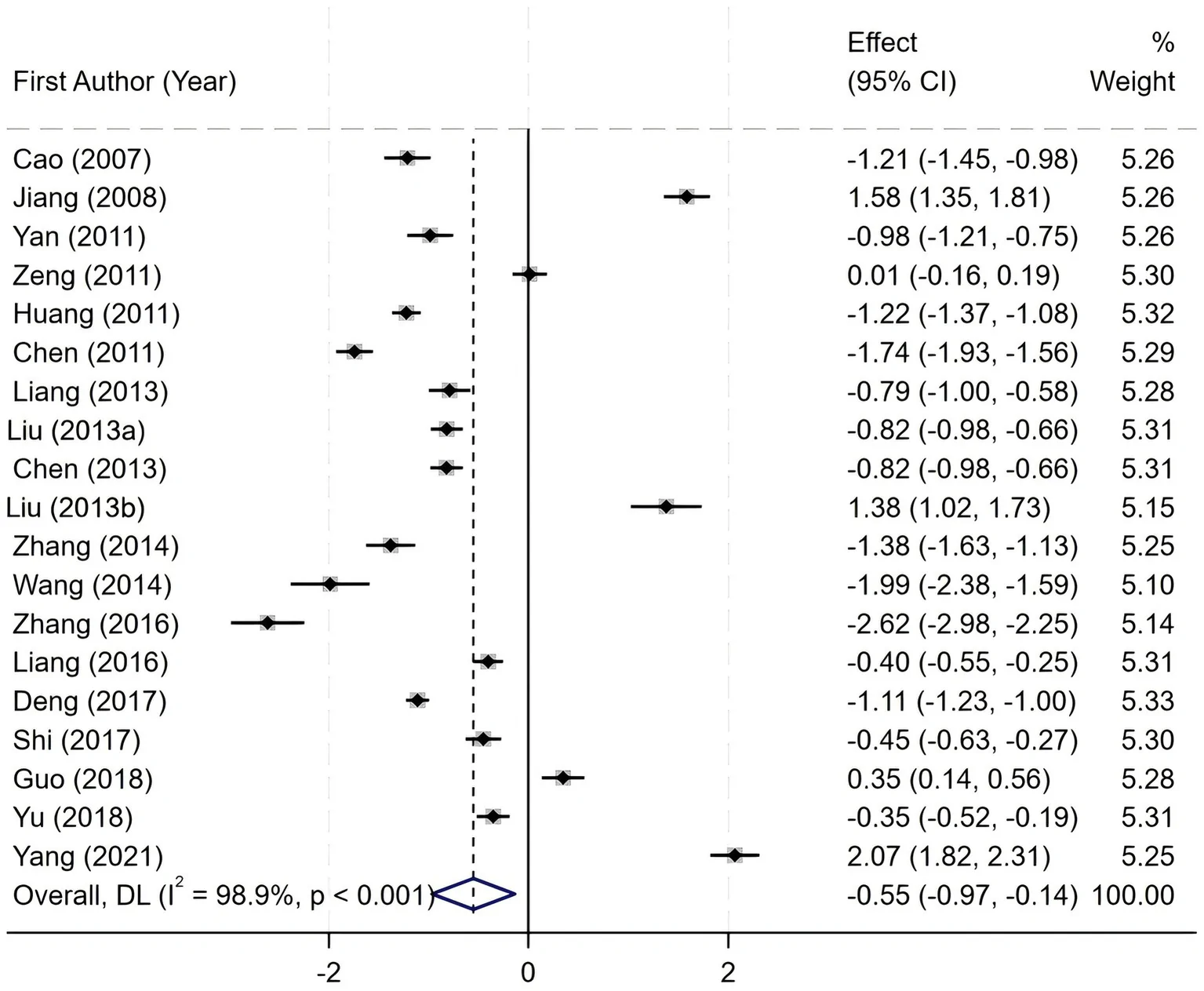
Forest plot of depersonalization.

As shown in Figure 3, random-effects meta-analysis yielded a pooled SMD of −1.30 (95% CI: −1.75 to −0.85) for depersonalization, which was statistically significant (Z = −5.652, *p* < 0.001). This represents a large negative effect size. The forest plot demonstrated that the majority of studies showed consistently negative SMDs with relatively narrow 95% confidence intervals, indicating markedly lower levels of depersonalization among Chinese university counselors. However, heterogeneity remained extremely high (*I*^2^ = 98.8%, Cochran’s Q-test *p* < 0.001), necessitating further moderator analyses to explore potential sources of variation.

### 3.4 Meta-analysis of reduced personal accomplishment

**Figure 4:**
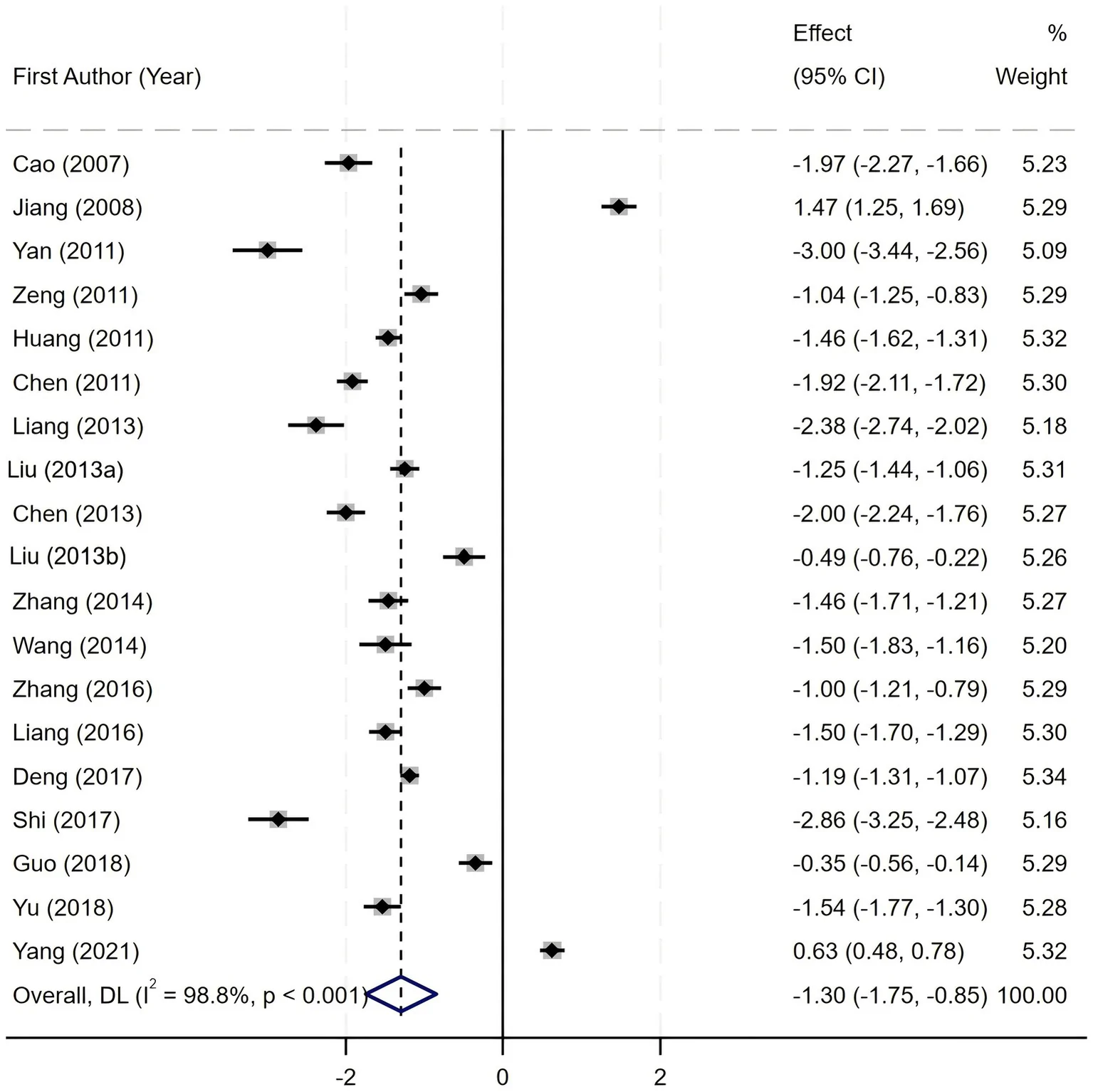
Forest plot of reduced personal accomplishment.

**Figure 5:**
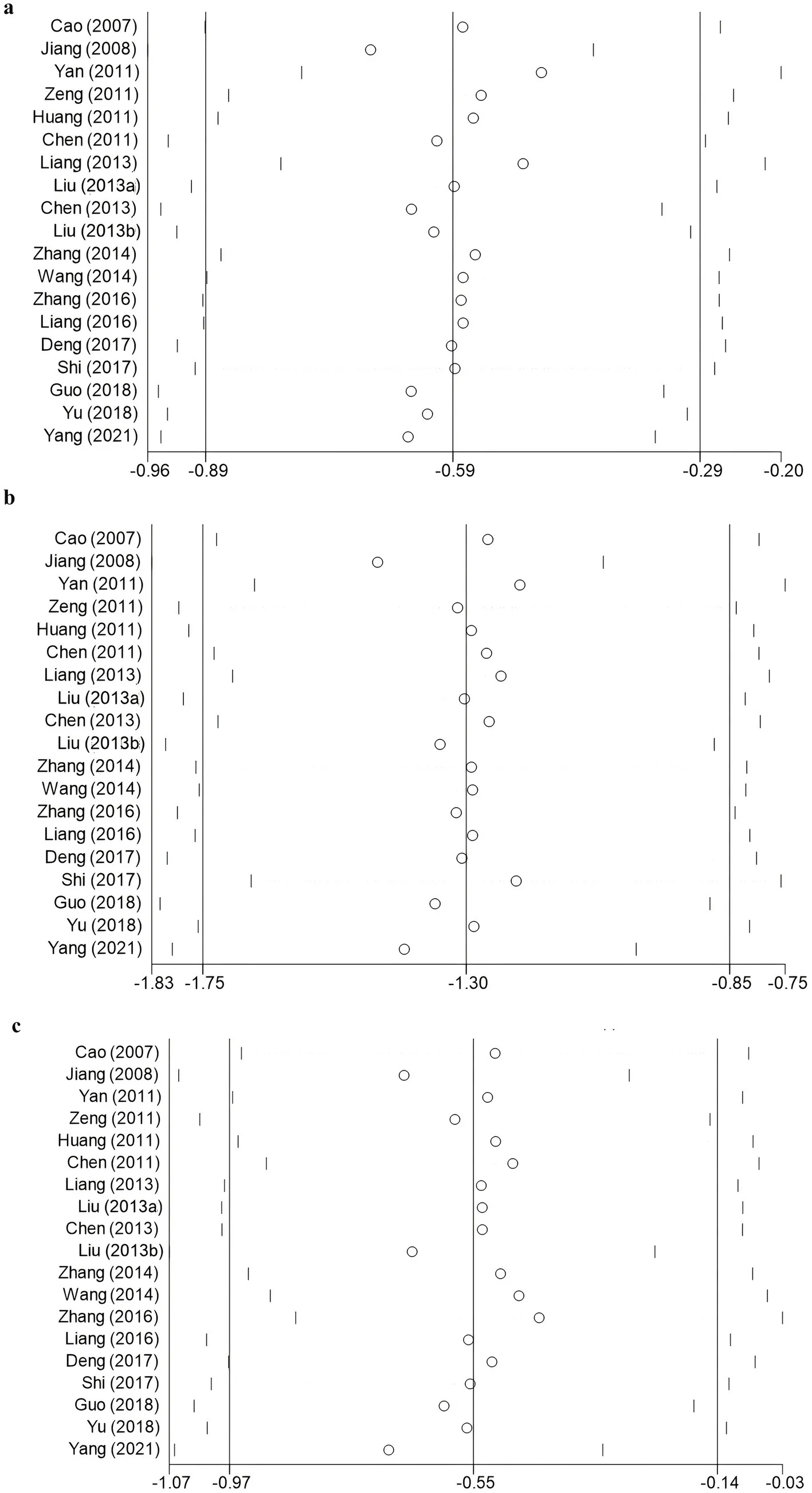
**(a)** Sensitivity analysis of emotional exhaustion. **(b)** Sensitivity analysis of depersonalization. **(c)** Sensitivity analysis of reduced personal achievement.

Random-effects meta-analysis (Figure 4) yielded a pooled SMD of −0.55 (95% CI: −0.97 to −0.14) for reduced personal accomplishment, which was statistically significant (*Z* = −2.603, *p* = 0.009). This corresponds to a moderate negative effect size. The forest plot demonstrated substantial heterogeneity (*I*^2^ = 98.9%), with considerable variation in effect sizes and relatively wide confidence intervals. Overall, according to Table 2, Chinese university counselors exhibited the lowest levels of depersonalization, followed by moderate levels of emotional exhaustion and reduced personal accomplishment. Emotional exhaustion and reduced personal accomplishment were the more prominent dimensions contributing to burnout in this population (Figure 5).

**Table 2:** The effect size of pooling across the three aspects of Pooled effect sizes across the three dimensions of job burnout.

Dimension	Number of studies	Sample size	Pooled SMD	95% CI	*Z* value	*p*-value	*I*^2^ (%)	Effect size
Emotional exhaustion	19	3,248	−0.59	(−0.89, −0.29)	−3.897	<0.001	98.2	Moderate
Depersonalization	19	3,248	−1.30	(−1.75, −0.85)	−5.652	<0.001	98.8	Large
Reduced personal accomplishment	19	3,248	−0.55	(−0.97, −0.14)	−2.603	0.009	98.9	Moderate

### 3.5 Caveat on the interpretation of global effects

Given the extreme heterogeneity observed across all three burnout dimensions (*I*^2^ > 98%), the pooled SMDs must be interpreted with utmost caution. These values should not be considered as precise, definitive estimates of the phenomenon for the entire population. Instead, they reflect general patterns and directional trends. The substantial variation underscores the influence of unmeasured contextual factors, emphasizing the need to focus on overarching patterns rather than the exact magnitude of the pooled effects.

### 3.6 Subgroup analysis

Subgroup analysis showed no statistically significant differences between the MBI-ES and MBI-GS series (all *p* > 0.05). This consistency aligns with previous validation studies suggesting that the core three-dimensional structure of the MBI remains stable across different versions within collectivist cultural contexts, indicating that both tools are highly interchangeable. Similarly, subgroup analysis by geographic region revealed no statistically significant differences (all *p* > 0.05). Given the lack of statistical significance, we must refrain from over-interpreting descriptive trends. The current evidence does not support the conclusion that regional disparities significantly modulate the overall burnout levels of Chinese university counselors (Table 3).

**Table 3:** Sub-groups of results.

Dimension	Subgroup	Group	Number of studies	Sample size	Pooled SMD (95% CI)	*I*^2^ (%)	Between-group *p*-value
Emotional exhaustion	Measurement tool	MBI-ES series	11	1,926	−0.59 (−0.90, −0.27)	97.3	0.828
		MBI-GS series	6	805	−0.69 (−1.51, 0.14)	98.9	
	Geographic region	Eastern China	8	1,344	−0.33 (−0.66, 0.00)	96.7	0.122
		Central-Western China	11	1,904	−0.79 (−1.27, −0.31)	98.7	
Depersonalization	Measurement tool	MBI-ES series	11	1,926	−1.26 (−1.79, −0.73)	98.8	0.903
		MBI-GS series	6	805	−1.25 (−2.33, −0.17)	99.4	
	Geographic region	Eastern China	8	1,344	−1.19 (−1.54, −0.85)	95.4	0.672
		Central-Western China	11	1,904	−1.38 (−2.15, −0.60)	99.2	
Reduced personal accomplishment	Measurement tool	MBI-ES series	11	1,926	−0.46 (−1.07, 0.16)	99.1	0.808
		MBI-GS series	6	805	−0.58 (−1.38, 0.22)	98.8	
	Geographic region	Eastern China	8	1,344	−0.69 (−1.24, −0.14)	98.5	0.587
		Central-Western China	11	1,904	−0.46 (−1.09, 0.18)	99.1	

### 3.7 Meta-regression analysis

Meta-regression was conducted to examine the effects of continuous moderators (Table 4). Publication year was a significant moderator for emotional exhaustion (*β* = 0.154, SE = 0.078, *p* = 0.048) and depersonalization (*β* = 0.15, SE = 0.064, *p* = 0.019), accounting for 9.5% of the heterogeneity. For depersonalization, when all moderators were entered simultaneously, they collectively explained 45.05% of the variance. The positive coefficients indicate that burnout levels (particularly emotional exhaustion and depersonalization) tended to increase over time from 2007 to 2021. No significant moderating effects were found for the proportion of male participants or log-transformed sample size (all *p* > 0.05) (Table 5).

**Table 4:** Meta-regression analysis results.

Dimension	Predictor variable	Coefficient (*β*)	Standard error	z	*p*-value	95% CI	*R*^2^ (%)
Emotional exhaustion	Publication year	0.154	0.078	1.98	0.048	(0.002, 0.307)	9.5
	Male proportion	0.018	0.021	0.84	0.402	(−0.024, 0.059)	
	Ln (sample size)	0.067	0.4	0.17	0.868	(−0.718, 0.851)	
Depersonalization	Publication year	0.15	0.064	2.35	0.019	(0.025, 0.275)	45.05
	Male proportion	−0.017	0.017	−0.96	0.335	(−0.051, 0.017)	
	ln (sample size)	−0.053	0.327	−0.16	0.871	(−0.693, 0.587)	
Reduced personal accomplishment	Publication year	0.187	0.119	1.57	0.116	(−0.046, 0.419)	2.63
	Male proportion	0.013	0.032	0.41	0.684	(−0.050, 0.076)	
	ln (sample size)	−0.554	0.609	−0.91	0.363	(−1.748, 0.639)	

**Table 5:** Sensitivity analysis results.

Dimension	Original pooled SMD (95% CI)	Pooled SMD after removing any one study (95% CI)
Emotional exhaustion	−0.59 (−0.89, −0.29)	−0.59 (−0.96, −0.29) ~ (−0.20)
Depersonalization	−1.30 (−1.75, −0.85)	−1.30 (−1.83, −0.75) ~ (−0.75)
Reduced personal accomplishment	−0.55 (−0.97, −0.14)	−0.55 (−1.07, −0.03) ~ (−0.03)

### 3.8 Sensitivity analysis

Sensitivity analyses using the leave-one-out method confirmed the stability of the results. Regardless of which individual study was excluded, the pooled SMDs and 95% confidence intervals for the three burnout dimensions remained materially unchanged and consistent with the primary analysis. This indicates that no single study exerted excessive influence on the overall conclusions.

### 3.9 Publication bias

**Figure 6:**
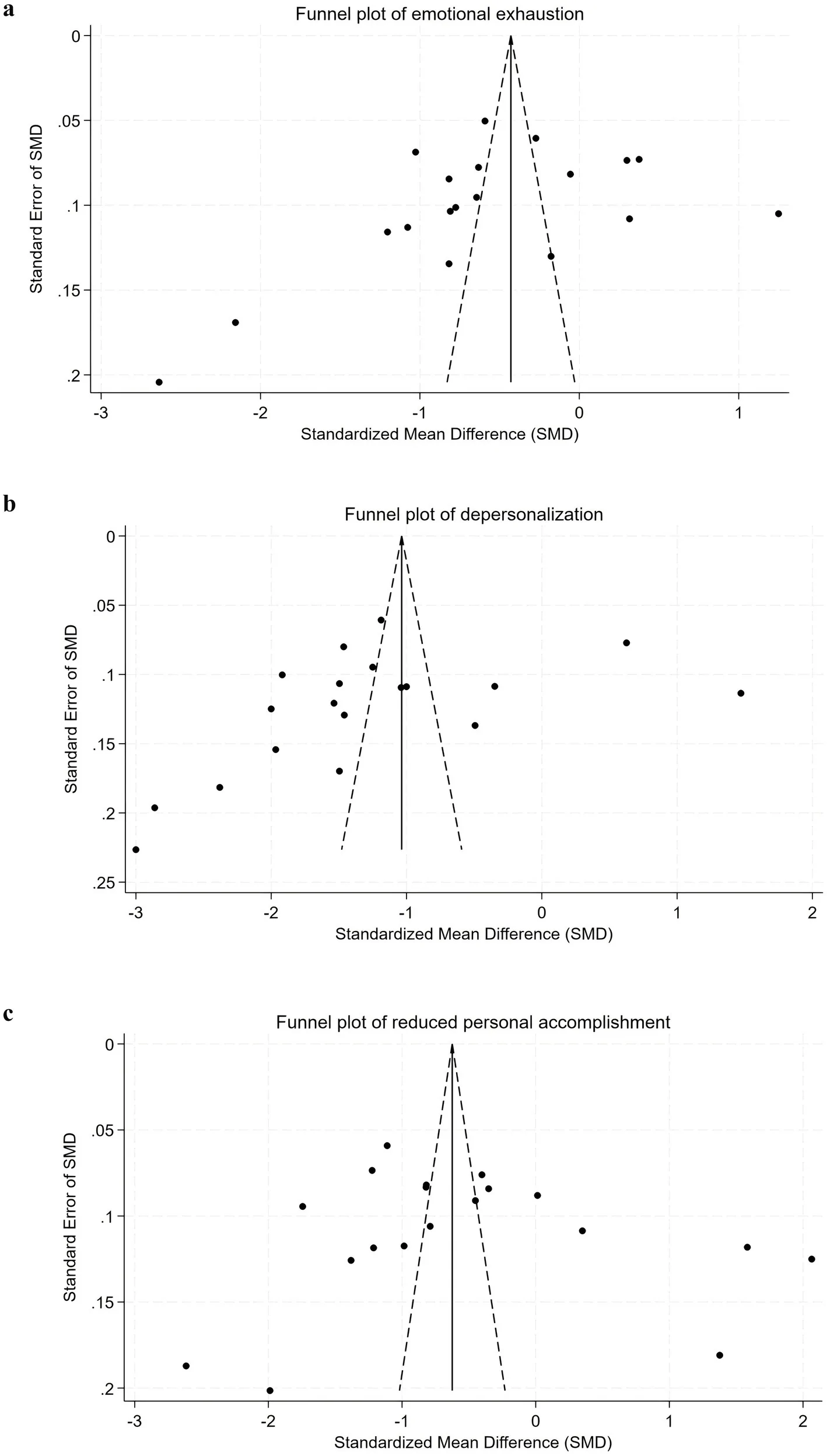
**(a)** Funnel plot for emotional exhaustion. **(b)** Funnel plot of depersonalization. **(c)** Funnel plot of reduced personal achievement.

Figure 6 funnel plot of three-dimensional data in Figures a, b and c. The Egger regression test showed some deviation from the asymmetry of the asymmetric function for emotional exhaustion (*z* = −3.14, *p* < 0.05) and depersonalization (*z* = −2.99, *p* = 0.0028); however, Begg’s rank correlation test did not show any deviation in any aspect. After trim-and-fill processing, five additional studies were imputed for depersonalization only; the adjusted pooled *SMD* value remained significantly negative at −0.899, falling inside its confidence interval [−1.404, −0.394], suggesting no considerable bias due to publication practices (Table 6).

**Table 6:** Test results for publication bias among dimensions of job burnout in university counselor(s).

Dimension	Number of studies	Egger’s test	Begg’s test	Trim-and-fill (studies added)	Original SMD (95% CI)	Adjusted SMD (95% CI)
Emotional Exhaustion	19	z = −3.14, *p* = 0.0017	*z* = −1.75, *p* = 0.0931	0	−0.596 (−0.989, −0.203)	—
Reduced Personal Accomplishment	19	z = −0.26, *p* = 0.7923	*z* = 0.42, *p* = 0.6746	0	−0.550 (−0.970, −0.140)	−0.550 (−0.970, −0.140)
Depersonalization	19	z = −2.99, *p* = 0.0028	*z* = −1.75, *p* = 0.0931	5	−1.299 (−1.784, −0.813)	−0.899 (−1.404, −0.394)

It is worth noting that Egger’s test and Begg’s test often yield inconsistent results in meta-analyses characterized by extreme heterogeneity (*I*^2^ ≥ 98%) and a limited number of included studies (*k* = 19). Egger’s regression test is generally more statistically sensitive to small-study effects and high between-study variance than Begg’s rank correlation test. In our study, the significant asymmetry detected by Egger’s test likely reflects the substantial clinical and methodological diversity among the primary studies, rather than pure publication bias. Furthermore, the trim-and-fill analysis for depersonalization imputed five hypothetical studies, yet the adjusted pooled SMD remained significantly negative (−0.899), indicating that the overall conclusions of this meta-analysis are robust and not fundamentally altered by potential publication bias.

## 4 Discussion

### 4.1 Main findings

A meta-analysis of 19 studies involving 3,248 Chinese university counselors revealed that depersonalization exhibited the most favorable profile among the three burnout dimensions, with the largest negative pooled SMD of −1.30 (large effect size). In contrast, emotional exhaustion (SMD = −0.59) and reduced personal accomplishment (SMD = −0.55) showed moderate negative effect sizes. These findings suggest that, although Chinese university counselors experience moderate levels of emotional exhaustion and reduced personal accomplishment under work pressure, they generally maintain relatively appropriate emotional responses and a low level of depersonalization/cynicism.

This pattern differs somewhat from findings in some Western contexts, where student affairs professionals and academic advisors often report higher emotional exhaustion (Jolicoeur et al., 2024; Jolicoeur, 2025; O’Rourke, 2024; Walker, 2023). The notably lower depersonalization among Chinese counselors may be explained by several cultural and contextual factors. First, the strong national emphasis on moral education and “cultivating people” fosters a deep sense of professional responsibility. Second, counselors maintain frequent and close daily contact with students. Third, the collectivist cultural orientation strengthens professional identity and sense of mission. These factors may collectively buffer against emotional stress and arousal, which are more commonly reported in Western studies that emphasize individualized professional roles and transactional interactions (Chessman, 2021; De Sawal et al., 2022; Wang, 2014). Future cross-cultural research is needed to further explore these protective mechanisms and develop culturally tailored interventions to address burnout in higher education settings.

### 4.2 Implications of subgroup analysis

The subgroup analyses in our study revealed no statistically significant differences in burnout levels across different measurement tools (MBI-ES vs. MBI-GS) or geographic regions (Eastern vs. Central-Western China). While null findings in the context of extreme heterogeneity (*I*^2^ > 98%) must be interpreted with caution, potentially reflecting limited statistical power for between-group comparisons, they still offer valuable theoretical insights into the unique professional context of Chinese university counselors.

First, the interchangeability of the MBI-ES and MBI-GS in this population suggests that the core three-dimensional structure of burnout is robust across different occupational framings. Chinese counselors hold a dual identity as both ideological and political educators and daily life administrators. Consequently, whether the assessment emphasizes the educational context (MBI-ES) or a general occupational setting (MBI-GS), it effectively captures the same underlying psychological exhaustion and relational detachment. This cross-version consistency validates the applicability of the MBI framework within China’s higher education student affairs system.

Second, regarding the geographic regions, the lack of significant regional disparities is intriguing given the well-documented economic inequalities between coastal and inland China. From a theoretical perspective, this finding may tentatively suggest that the core occupational demands of Chinese counselors create a “common baseline” of stress that transcends regional economic boundaries. Unlike general corporate employees whose burnout might be heavily modulated by regional salary or resource disparities, Chinese counselors are bound by a unified national professional identity and standardized ideological mandates (e.g., the Ministry of Education’s Order No. 43). The universal nature of their core tasks—such as 24/7 crisis intervention, ideological guidance, and the intense emotional labor of student mental health management—may act as a dominant stress (Li et al., 2019). Therefore, it is plausible that these shared, structurally identical professional demands outweigh the moderating effects of regional economic development, resulting in a broadly consistent burnout profile across the country. Future studies utilizing multilevel modeling are needed to empirically verify how institutional resources interact with these universal role demands across different regions.

### 4.3 Interpretation of meta-regression

Meta-regression analysis showed a significant positive relationship between the publication year effect and emotional exhaustion (*β* = 0.154, *p* < 0.05) and depersonalization (*β* = 0.150, *p* < 0.05). Based on this, we can know that since 2007–2021, the degree of burnout among them has gradually increased. This is mostly due to the continuous introduction of country-wide policies, namely, the 2014 Interim Standards for university counselor professional competence (Ministry of Education of the People’s Republic of China, 2014) and the 2017 Ministry of Education order no. 43 (Ministry of Education of the People’s Republic of China, 2017), which emphasized teacher qualification certificates and training courses more, along with teaching quality evaluation and welfare systems.

Internationally, the approaches to addressing these issues also provide valuable insights. For instance, American colleges vary in the time taken to implement staff health improvement plans, organizational reforms to improve the work environment, and particular burnout prevention measures (Jolicoeur, 2025; Mullen et al., 2018). The effectiveness of these international practices highlights that establishing systemic organizational support is crucial for mitigating stress-related issues. However, it is also evident that factors beyond national culture, such as specific institutional environments and societal expectations, significantly shape how counselors perceive their roles and experience burnout.

### 4.4 Considerations regarding high heterogeneity

There was extreme heterogeneity (*I*^2^ > 98%) among the three types of burnout across all meta-analyses based on psychological and educational data from various organizations and institutions with adjustments. Subgroup analysis according to the instrument used for measurement, regional differences, and publication year failed to fully address all aspects of variation; that is, a significant portion of the variation remained unexplained. Potential sources of bias could be variations in institution category (e.g., undergraduate university vs. Higher Vocational College), counselor’s years of service, number of students per counselor, work independence, leadership encouragement, and other factors not continuously provided by both primary research. Given that the very high I2 scores are related to multiple aspects of burnout, which may involve general occupational stress factors for workers in various industries or culture-specific context variables in Chinese higher education environments (Gu et al., 2019). Other scholars will learn to use multi-level modeling or add other regulators, such as workload intensity and sense of organizational support, in the future to further explore this phenomenon under different circumstances (Mullen et al., 2018; Jolicoeur, 2025). The researcher did not expand the range of the pooled results and did not account for the differences among individuals in the various studies or biases caused by cross-sectional design.

### 4.5 Limitations

However, this meta-analysis has several critical limitations that warrant a cautious interpretation of the findings. First, although extreme heterogeneity (*I*^2^ > 98%) was observed across all three burnout dimensions, our meta-analytic framework was limited by the aggregated nature of the extracted data. We were unable to conduct an Individual Participant Data (IPD) meta-analysis, which restricted our ability to explore specific individual-level or institutional moderators, such as exact workload and institutional support, that might explain this variance. Second, our reliance on the cross-sectional designs and non-probabilistic convenience samples of the primary literature precludes us from making causal inferences regarding the trajectories of burnout and limits the national representations of the findings to the broader population of Chinese university counselors. Third, there is considerable variability in the methodological quality of the included studies; although we excluded low-quality studies, the inherent limitations of these primary studies may introduce selection bias and affect the precision of the pooled estimates. Fourth, although the literature search extended to March 2026, no new cross-sectional studies meeting the strict inclusion criteria were identified after 2022, limiting the timeliness of the findings regarding post-pandemic changes. Finally, despite comprehensive searches in both Chinese and English databases, the possibility of language or publication bias cannot be completely excluded. Future research must prioritize large-scale, nationally representative longitudinal studies with detailed individual-level data to provide more robust estimates and explore the causal mechanisms of burnout in this population.

### 4.6 Practical implications

University psychological services should clarify career development pathways for university counselors. To maintain the currently low level of depersonalization, institutions are advised to strengthen the teacher-student bond. Regular burnout assessments using validated instruments (e.g., MBI-ES or MBI-GS) are recommended to enable objective and timely monitoring.

Mechanism-based interventions should include emotional labor management training, psychological capital development programs, mindfulness-based activities, and the establishment of peer support systems. These measures aim to foster a sustainable and supportive work environment. Valuable insights can also be drawn from international best practices. For instance, student affairs departments in U.S. universities have effectively reduced staff burnout through flexible working hours, reasonable workload management, formal peer supervision mechanisms, regular burnout screening, and organizational reforms that enhance professional recognition and work-life balance (Jolicoeur, 2025).

Chinese universities should adopt context-specific management strategies tailored to their actual conditions. By integrating multi-level interventions based on the Job Demands-Resources (JD-R) model, encompassing individual skill enhancement, organizational capacity building, and policy support, universities can improve counselors’ professional well-being, reduce staff turnover, and ultimately enhance the quality of holistic student development.

## 5 Conclusion

This meta-analysis, based on 19 studies involving 3,248 Chinese university counselors, found that depersonalization was the most favorable dimension among the three burnout dimensions, with a large negative pooled SMD of −1.30. Emotional exhaustion (SMD = −0.59) and reduced personal accomplishment (SMD = −0.55) showed moderate negative effect sizes. These results indicate that Chinese university counselors generally experience relatively low levels of depersonalization or cynicism, while emotional exhaustion and reduced personal accomplishment remain moderate concerns. Meta-regression further revealed that burnout levels in emotional exhaustion and depersonalization have gradually increased from 2007 to 2021. To effectively address burnout, universities should strengthen the teacher-student bond, optimize workload distribution, and provide systematic mental health support. It is recommended to establish clear career development pathways, implement emotional labor management training, and develop psychological capital enhancement programs. Drawing on both international best practices and China’s policy context, multi-level interventions based on the Job Demands-Resources (JD-R) model can significantly improve counselors’ professional well-being, reduce turnover rates, and ultimately promote the all-round development of students by integrating individual skill development, organizational support, and policy guidance.
